# Systematic Microwave-Assisted Postsynthesis of Mn-Doped Cesium Lead Halide Perovskites with Improved Color-Tunable Luminescence and Stability

**DOI:** 10.3390/nano12152535

**Published:** 2022-07-23

**Authors:** Yaheng Zhang, Chao Fan, Jianghong Tang, Gaoming Huang, Xinfa Qiang, Yu Fu, Wenjuan Zhou, Juan Wu, Shouqiang Huang

**Affiliations:** 1 Jiangsu Key Laboratory of E-Waste Recycling, School of Chemistry and Environmental Engineering, Jiangsu University of Technology, Changzhou 213001, China; zhangyaheng@jsut.edu.cn (Y.Z.); chaofan19930703@163.com (C.F.); tjh01@jsut.edu.cn (J.T.); hanggaoming1122@163.com (G.H.); fuyu@jsut.edu.cn (Y.F.); 2019560060@jsut.edu.cn (W.Z.); jintanwujuan@163.com (J.W.); 2 Jiangsu Key Laboratory of Advanced Structural Materials and Application Technology, Nanjing Institute of Technology, Nanjing 211167, China; qiangxinfa@163.com

**Keywords:** microwave heating, postsynthetic doping strategy, Mn^2+^-doped cesium lead halide perovskite, photoluminescence, stability

## Abstract

The metal doping at the Pb^2+^ position provides improved luminescence performance for the cesium lead halide perovskites, and their fabrication methods assisted by microwave have attracted considerable attention due to the advantages of fast heating and low energy consumption. However, the postsynthetic doping strategy of the metal-doped perovskites driven by microwave heating still lacks systematic research. In this study, the assembly of CsPbBr_3_/CsPb_2_Br_5_ with a strong fluorescence peak at 523 nm is used as the CsPbBr_3_ precursor, and through the optimization of the postsynthetic conditions such as reaction temperatures, Mn^2+^/Pb^2+^ feeding ratios, and Mn^2+^ sources, the optimum Mn^2+^-doped product (CsPb(Cl/Br)_3_:Mn) is achieved. The exciton fluorescence peak of CsPb(Cl/Br)_3_:Mn is blueshifted to 437 nm, and an obvious fluorescence peak attributing to the doped Mn^2+^ ions at 597 nm is obtained. Both the CsPbBr_3_ precursor and CsPb(Cl/Br)_3_:Mn have high PLQY and stability because there are CsPb_2_Br_5_ microcubic crystals to well disperse and embed the CsPbBr_3_ nanocrystals (NCs) in the precursor, and after Mn^2+^-doping, this structure is maintained to form CsPb(Cl/Br)_3_:Mn NCs on the surface of their microcrystals. The exploration of preparation parameters in the microwave-assisted method provides insights into the enhanced color-tunable luminescence of the metal-doped perovskite materials.

## 1. Introduction

Perovskite solar cells were rated as one of the “Top Ten Scientific Breakthroughs” by Science in 2013, leading to the emergence of the “perovskite craze” in the world. However, its key core material, organic-inorganic hybrid perovskite, has poor stability, and it is easy to degrade in a short time under the condition of exposure to light, heat, water, or oxygen, which is seriously restricted its popularization and application [1,2]. In this context, people began to pay attention to all-inorganic trihalogen perovskite materials (e.g., CsPbBr_3_), which have similar excellent photoelectric performance and better stability. Since the first report of all-inorganic perovskite nanocrystalline materials in January 2015 [3], CsPbX_3_ (X = Cl, Br, I, or their mixture) nanocrystals (NCs) have attracted much attention due to their core advantages such as excellent photoelectric properties, broad application prospects and simple preparation process [4,5,6,7]. Related research has mushroomed and shown a rapid growth trend, which has become a new research frontier in the field of photoelectric materials. However, the extensive use of toxic Pb^2+^ in perovskite NCs seriously limits their practical application. At the same time, CsPbX_3_ also faces the urgent need to adjust or improve its performance, especially luminescence performance, to adapt to its photoelectric application or expand its application scope.

There are Cs^+^, X^−^ and Pb^2+^ in CsPbX_3_ lattice. Researches show that compared with X^−^ and Pb^2+^, there is little influence on its luminescence properties by doping other ions at Cs^+^ [8,9]. By doping different halogen ions to the position of X^−^, the band gap could be changed, and the luminescence peaks of CsPbX_3_ can be regulated to some extent [3,10]. The 6s and 6p orbitals of Pb^2+^ contribute greatly to the formation of valence band top and conduction band bottom, and the lattice position of Pb^2+^ plays an extremely important role in determining its photoelectric properties [11]. Accordingly, it is desirable to conduct effective metal ion doping at the Pb^2+^ position [12]. That is, the dopants replace part of Pb^2+^ ions rather than adsorbing on the surface of the NCs. Mn^2+^ is one of the most popular dopants. The main reasons are as follows: firstly, Mn^2+^ has the same charge number as Pb^2+^ and a smaller ionic radius, which is conducive to doping [13,14,15,16,17]; Secondly, Mn^2+^ can be used as a tracer ion [14,18]. The successful doping and doping site can be judged by its emission or electron paramagnetic resonance spectra [19,20]. Moreover, its stokes displacement is large, and its self-absorption effect is small. Mn^2+^ is also an exciton coupling agent, which can obtain energy from the matrix (known as host) to exhibit its characteristic luminescence [21,22]. The driving force is another important factor affecting effective doping. The doping driving force has at least two functions: one is to interrupt part of the Pb-X bonds in the CsPbX_3_ lattice, and the other is to promote the diffusion of dopants, such as Mn^2+^ ions, and eventually occupy part of the Pb^2+^ sites. At present, hot injection is the most common doping method in preparation of CsPbX_3_ NCs [23,24,25]. In addition, microwave, ultrasound or pressure can also drive impurity ions into CsPbX_3_ lattice [26,27]. Among various synthesis methods, the microwave-assisted method has attracted much attention, wherein microwave radiation is used as a relatively fast energy input source to drive the reaction. Compared with other heating methods, microwave heating has the advantages of more uniform, fast reaction, low energy consumption and selective heating, which are conducive to the nucleation of CsPbX_3_ NCs.

There are two main doping strategies at the lattice site of Pb^2+^: in situ doping and postsynthetic doping. The former is the addition of dopants, Cs^+^ and Pb^2+^ sources into the reaction system [28,29,30], belonging to nucleation doping. The latter is based on the dopant and the pre-prepared CsPbX_3_ NCs as the starting materials, and the doped CsPbX_3_ NCs are obtained after this post-treatment [31,32] belonging to growth doping. The process of nucleation doping to form CsPbCl_3_:Mn^2+^ NCs under thermal driving is fast, completed in about 5 s, and the postsynthetic doping strategy to form CsPbCl_3_:Mn^2+^ NCs is slow but easy to control [33]. At present, most of CsPbX_3_:M^Z+^ (M^Z+^ represents the doped metal ions) NCs are synthesized by in situ doping [12,23,24,28,29]. It was also reported that CsPbX_3_: M^Z+^ NCs can also be prepared by a postsynthetic doping strategy. For example, Donegá et al. [31] synthesized CsPbBr_3_:M^2+^ NCs by mixing MBr_2_ (M = Zn^2+^, Cd^2+^, Sn^2+^) and the benzene dispersion of CsPbBr_3_ NCs at room temperature. Xu et al. [33] found that CsPbCl_3_:Mn^2+^ NCs could be formed by mixing MnCl_2_ dispersion (dispersed in 1-octadecene (ODE)), oleylamine (OLA), and oleic acid (OA) with CsPbCl_3_ nanocrystals at 150 °C and stirring. Wang et al. [32] reported that Mn^2+^-doped NCs could be obtained by mixing the N, N-dimethylformamide dispersion of MnCl_2_ with the benzene dispersion of CsPbBr_3_ NCs at 20 °C and stirring violently. Xia et al. [34] mixed and stirred the hexane dispersion of CsPbBr_3_ and CsPbCl_3_:Mn^2+^ NCs at room temperature to obtain CsPb(Cl, Br)_3_:Mn^2+^ NCs. In the application of microwave heating, Liu et al. [26] put the reaction mixture, which included cesium acetate, PbCl_2_, MnCl_2_, ODE, bis(2,4,4trimethylpentyl) phosphinic acid (TMPPA), and OLA into a microwave oven, and CsPbCl_3_:Mn^2+^ was obtained after 20 min reaction at 800 W by this one-step microwave-assisted preparation method. The maximum photoluminescence quantum yield (PLQY) belongs to the CsPb_0_._79_Mn_0_._21_Cl_3_ NCs with a value of 26%. Yang et al. [35] synthesized CsPb(Br,Cl)_3_:Mn^2+^ NCs without inert atmosphere at room temperature by a microwave-assisted method, which is also attributed to the in situ doping method. However, it is noted that, the systematic research on postsynthetic doping of Mn^2+^ into CsPbX_3_ NCs driven by microwave is still very limited, which needs further investigation.

In this study, the assembly of CsPbBr_3_/CsPb_2_Br_5_ with excellent luminescent properties was initially obtained by screening the synthesis conditions under microwave-driven reactions, and the optimum assembly was used as the CsPbBr_3_ precursor. In comparison with a domestic microwave oven, the reaction temperature and pressure of the mixture can be controlled automatically in a closed reaction bottle with high safety. On the basis of the CsPbBr_3_ precursor, the Mn-doped lead halide perovskites with excellent luminescence were obtained by investigating the reaction temperatures, times, Mn^2+^ doping ratios, Mn^2+^ sources, and the volume ratios of ODE/diethylene glycol butyl ether (DGBE). The structure and PL emission spectra of the Mn^2+^ doped products are investigated to obtain the best condition. From the comparison between the CsPbBr_3_ precursor and the optimum Mn^2+^ doped product in PL emission, morphology, composition, and stability, the mechanism for the improved PL quantum yields (PLQYs) and stabilities are discussed. Under UV excitation, the Mn^2+^ doped products not only exhibit the exciton luminescence of perovskite (also known as band edge luminescence) but also the luminescence of Mn^2+^ ions, realizing the tunable luminescence color.

## 2. Materials and Methods

### 2.1. Chemicals and Materials

Cs_2_CO_3_ (cesium carbonate, 99.9%), PbBr_2_ (lead bromide, 99.0%), ODE (90%), OA (90%), OLA (90%), DGBE (99.0%), MnCl_2_·4H_2_O (manganese chloride tetrahydrate, 99.0%), MnBr_2_·4H_2_O (manganese bromide hydrate, 98.0%), MnSO_4_·H_2_O (manganese sulfate monohydrate, 99.0%), C_4_H_6_MnO_4_·4H_2_O (manganese acetate tetrahydrate, 99.0%), and C_10_H_14_MnO_4_ (manganese acetylacetonate, 99.5%) were purchased from Shanghai Makclin Biochemical Co., Ltd. (Shanghai, China). Cyclohexane (99.7%) and ethyl acetate (99.5%) were purchased from China National Pharmaceutical Group Corporation (Beijing, China).

### 2.2. Preparation of the CsPbBr_3_ Precursor

Firstly, Cs_2_CO_3_ (0.0326 g), PbBr_2_ (0.1668 g), DGBE (5.00 mL), ODE (5.00 mL), OA (0.50 mL), and OLA (0.50 mL) were put into the microwave flask. Then, the stirrer was added, and put the microwave flask into the microwave instrument (NOVA-2S, China). The pristine CsPbBr_3_ was synthesized by temperature programming (heating from room temperature to 150 °C for 3 min and holding for 15 min). When the samples were cooled to 71 °C, the microwave flasks were taken out from the microwave instrument. After cooling to room temperature with ice water, the mixtures were centrifuged at 8500 rpm/min for 8 min, and then the solids were washed twice with ethyl acetate (centrifugation). All the obtained CsPbBr_3_ precipitates were temporarily placed in the centrifuge tube. To find the optimum condition, other kinds of CsPbBr_3_ were also prepared with different mole ratios of Cs_2_CO_3_/PbBr_2_ (0.1/0.25~0.1/0.5), temperatures (100~200 °C), and volume ratios of ODE/DGBE (5/2.5~5/12.5).

### 2.3. Postsynthesis of Mn^2+^ Doped Perovskites

DGBE, ODE (5 mL), OA (0.50 mL), OLA (0.50 mL), and MnCl_2_·4H_2_O (0.90 mmol) were added into the above centrifuge tube containing the CsPbBr_3_ precursor. After the mixture was uniformly dispersed, it was transferred to the microwave flask. The stirrer was added to the flask, which was then put into the microwave instrument for reaction. The process is the same as that mentioned above with different temperatures, times, Mn^2+^ doping ratios, and volume ratios of ODE/DGBE. Finally, the products were cooled with ice water, centrifuged at 8500 rpm/min for 8 min, and washed twice with ethyl acetate, which was then stored in cyclohexane. Meanwhile, other Mn^2+^ sources of MnBr_2_·4H_2_O, MnSO_4_·H_2_O, C_4_H_6_MnO_4_·4H_2_O, and C_10_H_14_MnO_4_ were also chosen to substitute MnCl_2_·4H_2_O to prepare the products for comparison.

### 2.4. Characterizations

The PL spectra, PLQYs, and stability measurements of the CsPbBr_3_ precursor and Mn^2+^-doped products cyclohexane solutions (all the optical absorbances were adjusted to the same before detection) were tested by using a fluorescence spectrophotometer (FluoroMax Plus, Horiba Scientific), and the excitation wavelength was 365 nm. For the test of PLQY, an integrating sphere accessory was used, and the parameters of 2.4 nm slit width and 1s integration time were used; the excitation wavelength was 365 nm, and the scanning range of emission spectrum was 415–710 nm; cyclohexane was taken as a blank sample, the product concentration was adjusted to make the absorbance value ≤ 0.05; the PLQY calculations were performed using the Horiba Scientific FluorEssence software, and the integrating sphere calibration file was selected in the software; the sample spectrum and blank spectrum were imported respectively, and the fitting ranges of excitation and emission wavelengths were adjusted to obtain the PLQY value. The PL decay curves were recorded on the same fluorescence spectrophotometer, where a 370 nm NanoLED pulsed source was used as the excitation source. The UV-vis absorption spectra were detected on a UV1800PC spectrophotometer (Shanghai Jinghua Instruments, Room 1310, Building 4, Songjiang Wanda Plaza, Shanghai, China). The X-ray diffraction (XRD) investigations were performed using a Philips X’Pert Powder Diffractometer system with Cu K_α_ radiation. The morphologies and compositions were recorded by the Zeiss Sigma 500 field emission scanning electron microscope (SEM) and the JEOL JEM 2100F transmission electron microscope (TEM). The surface compositions of the samples were obtained by the K-Alpha + X-ray photoelectron spectroscopy system (XPS, Thermo Fisher Scientific, Building 3& 6& 7, 27 Xinjinqiao Road, Pudong New Area, Shanghai, China). Electron paramagnetic resonance (EPR) was measured on a Bruker A300 spectrometer. Fourier transform infrared (FTIR) spectra were recorded on a Nicolet 6700 spectrometer.

## 3. Results and Discussion

### 3.1. Optimization of the Synthesis of the CsPbBr_3_ Precursor

The pristine CsPbBr_3_ products are first prepared by a microwave method, wherein the feed mole ratios of Cs_2_CO_3_/PbBr_2_, temperatures of microwave reaction, and the volume ratios of ODE/DGBE added are discussed to obtain products with better luminous performance. As shown in Figure S1, when the mole ratio of Cs_2_CO_3_/PbBr_2_ is 0.1/0.45, the product shows excellent PL emission intensity, indicating a suitable feed ratio is one of the important factors for obtaining optimum products. The reaction temperatures vary from 100 to 200 °C, and the highest PL intensity is exhibited at 150 °C (Figure S2). To absorb microwaves better and achieve a higher microwave reaction effect, the polar substance content of DGBE in the system needs to be concerned. In the experiments, the addition of ODE is constant, while the volume ratios of ODE/DGBE are changed. When the ratio reaches 5/5, the product has the strongest PL emission (Figure S3). On the basis of the above optimized parameters, the best product is obtained under the conditions of Cs_2_CO_3_/PbBr_2_ = 0.1/0.45, temperature of 150 °C, and ODE/DGBE = 5/5.

**Figure 1:**
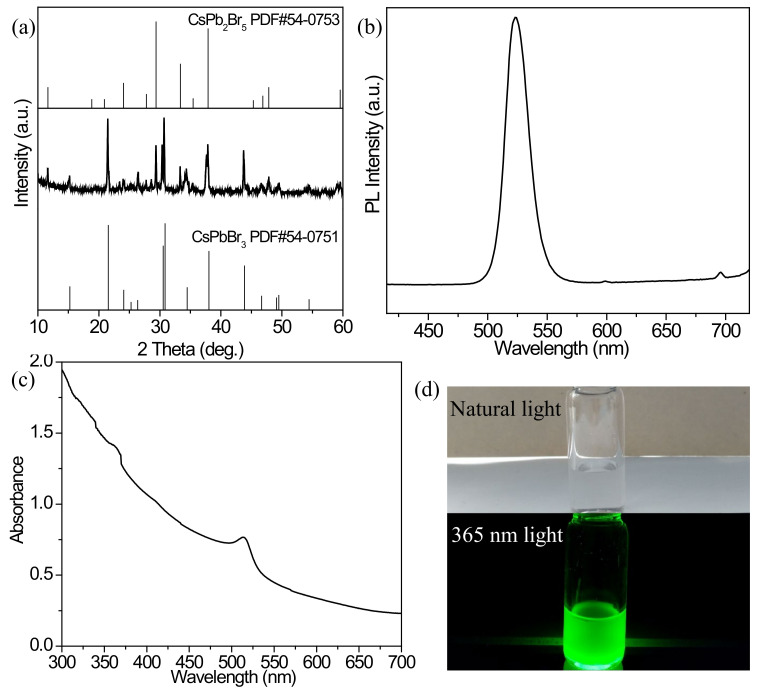
(**a**) XRD pattern, (**b**) PL emission and (**c**) UV-vis absorption spectra of the best pristine CsPbBr_3_ achieved under the conditions of Cs_2_CO_3_/PbBr_2_ = 0.1/0.45, temperature of 150 °C and ODE/DGBE = 5:5. (**d**) Optical images of the cyclohexane solution containing the best pristine CsPbBr_3_ under the environments of natural and 365 nm light.

Figure 1a shows the XRD pattern of the best product. It can be found that most of the diffraction peaks are consistent with the standard card of PDF#54-0751, which belongs to the monoclinic phase of CsPbBr_3_, and the 2θ at 21.55° and 30.84° correspond to the (010) and (002) crystal planes of CsPbBr_3_, respectively. In addition, there are also diffraction peaks assigned to the standard card of PDF#54-0753, indicating that there is a small amount of CsPb_2_Br_5_ in the pristine CsPbBr_3_ product. The obvious diffraction peaks indicate their suitable crystallinity. Figure 1b shows the PL emission spectrum of the pristine CsPbBr_3_ excited at 365 nm, and a narrow, strong fluorescence emission peak at 523 nm with 18 nm half-peak width (FWHM) is present. Figure 1c shows the UV-vis absorption spectrum of the pristine CsPbBr_3_, and the first excitonic absorption peak is located at 515 nm. The photographs of the pristine CsPbBr_3_ respectively excited under natural and 365 nm UV light are given in Figure 1d, and a bright green color is exhibited with a high PLQY of about 93%. This assembly of CsPbBr_3_ containing CsPb_2_Br_5_ is used as the precursor for the synthesis of the Mn^2+^-doped products.

### 3.2. Postsynthesis of the Mn^2+^ Doped Products

#### 3.2.1. Effect of Reaction Temperature

**Figure 2:**
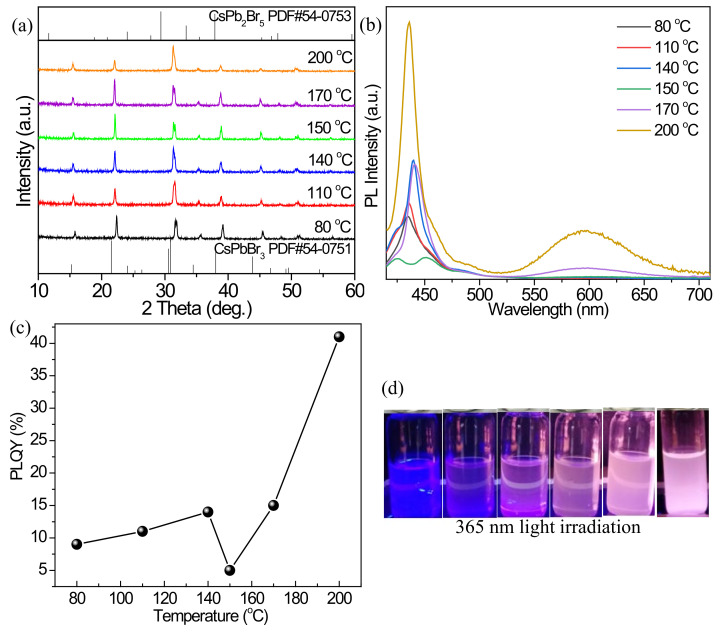
(**a**) XRD patterns, (**b**) PL emission spectra, and (**c**) PLQYs of the Mn^2+^-doped products fabricated at different reaction temperatures. (**d**) Optical images of the cyclohexane solutions containing the Mn^2+^-doped products under the environment of 365 nm light: from left to right, the reaction temperatures are 80, 110, 140, 150, 170, and 200 °C, respectively.

On the basis of the CsPbBr_3_ precursor with excellent luminescent properties, the Mn^2+^ post-doping experiments are carried out. In a chemical reaction, thermodynamic and kinetic factors determine the reaction rate, yield, and morphology of the products, so the reaction temperature is an important factor for the doping experiments. The doped inorganic perovskites are synthesized by doping Mn^2+^ into the CsPbBr_3_ precursor, and MnCl_2_·4H_2_O is selected as the Mn^2+^ source. The effect of reaction temperature on the Mn^2+^ doped products is investigated under the Mn^2+^/Pb^2+^ feed ratio of 1/1, ODE/DGBE volume ratio of 5/5, and reaction time of 15 min. Figure 2a shows the XRD patterns of the products synthesized at different temperatures. In comparison with the standard card PDF#54-0751 of CsPbBr_3_, the diffraction peaks of the products in this system all shift to higher angles. The reason for this phenomenon is that parts of Pb^2+^ and Br^−^ in the CsPbBr_3_ precursor have been replaced by Mn^2+^ and Cl^−^, respectively. Because the ionic radius of Mn^2+^ is smaller than that of Pb^2+^ and the ionic radius of Cl^−^ is also smaller than that of Br^−^ [12,32,36], the substitution of this cation and anion causes perovskite lattice shrinkage. As the increase in reaction temperatures, some diffraction peaks are gradually enhanced, such as the peak at 2θ = 31.5°, indicating the suitable crystallinity of the Mn^2+^ doped products. Furthermore, the diffraction peaks of CsPb_2_Br_5_ corresponding to PDF#54-0753 almost disappeared even at the lowest temperature of 80 °C, suggesting the products obtained mainly consisted of the Mn^2+^ doped CsPbBr_3_. It is noted that, through the replacement of Pb^2+^ in CsPb_2_Br_5_ by Mn^2+^, the phases of Cs(Pb/Mn)Br_5_ and CsMn_2_Br_5_ (1:2:5) are difficult to form, but CsMnBr_3_ (1:1:3) often tends to produce [24,37]. Accordingly, after the partial replacements of Pb^2+^ by Mn^2+^ and Br^−^ by Cl^−^, the 1:2:5 structure is destroyed, and instead, the 1:1:3 structure of Cs(Mn/Pb)(Cl/Br)_3_ is preferred to generate.

Figure 2b is the PL emission spectra of the Mn^2+^ doped products synthesized in the reaction temperature system. Compared with the CsPbBr_3_ precursor, the exciton emission peaks of the products are blueshifted from 523 nm to about 440 nm (Figure S4a), which is caused by the partial replacement of Br^−^ ions in the CsPbBr_3_ precursor by Cl^−^ ions of MnCl_2_. At 80 °C, the Cl^−^ ions have entered the CsPbBr_3_ precursor, and a large blueshift of the exciton emission peak is present. As the temperature increases from 80 to 140 °C, the exciton fluorescence peak intensity is first enhanced and then decreased, while there is almost no Mn^2+^-related fluorescence peak. At the higher temperature of 170 °C, an obvious Mn^2+^ characteristic fluorescence emission peak appears near 600 nm (Figure S4b), indicating that Mn^2+^ ions have begun to be doped into the CsPbBr_3_ precursor. When the reaction temperature reaches 200 °C (the highest temperature of the microwave instrument can reach), the intensity of Mn^2+^ characteristic fluorescence emission peak is the largest, and the exciton energy transfer efficiency from excitons to Mn^2+^ is the highest. At this temperature, the exciton emission peak intensity is also obvious, and the calculated PLQY from both the exciton and Mn^2+^ emission peaks reaches 41% (Figure 2c), which is higher than those obtained at other temperatures, so 200 °C is a suitable reaction temperature for Mn^2+^ post-doping. Therefore, the Cl^−^ and Br^−^ can exchange at a lower temperature in the microwave reaction system, while, for the exchange of Pb^2+^ by Mn^2+^, it needs a higher temperature. The photographs of the doped products irradiated by natural and 365 nm UV light are given in Figure 2d. When the reaction temperature reaches 200 °C, very bright orange light is emitted from the Mn^2+^ doped product, which is very different from the products prepared at other reaction temperatures, indicating that Mn^2+^ ions have been effectively doped in the CsPbBr_3_ precursor at this condition.

#### 3.2.2. Effect of Reaction Time

In the growth process of crystals, the reaction time also has a great influence on the luminescence intensity of the samples. For example, if the crystal growth time is too short, the defects caused by incomplete growth would lead to the weakening of the luminescence intensity. In contrast, the long growth time may cause the agglomeration and stacking of crystals to form large particles, affecting the luminescence of the samples. Accordingly, the effect of reaction time on the doped products is investigated under the Mn^2+^ (MnCl_2_·4H_2_O)/Pb^2+^ feeding ratio of 1/1, ODE/DGBE volume ratio of 5/5, and reaction temperature of 200 °C.

**Figure 3:**
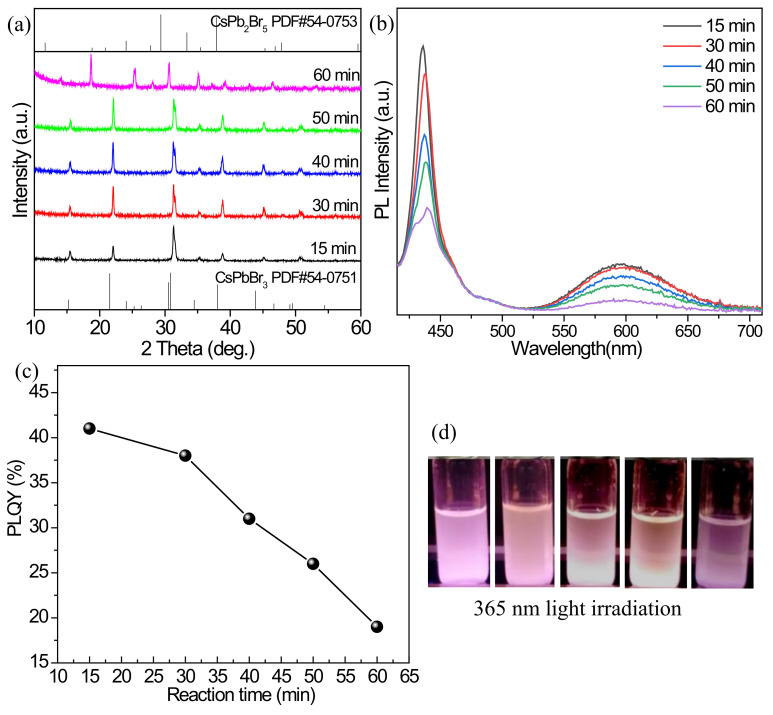
(**a**) XRD patterns, (**b**) PL emission spectra, and (**c**) PLQYs of the Mn^2+^-doped products fabricated with different reaction times. (**d**) Optical images of the cyclohexane solutions containing the Mn^2+^-doped products under the environment of 365 nm light: from left to right, the reaction times are 15, 30, 40, 50, and 60 min, respectively.

Figure 3a shows the XRD patterns of the Mn^2+^ doped products synthesized in the reaction time system. The diffraction peaks are generally shifted to higher angles (Figure S5) compared with the standard card PDF#54-0751 of CsPbBr_3_. The reason is the same as that of the temperature system, which is caused by the lattice shrinkage from the Mn^2+^ substitution. In addition, with the increase in reaction time, the crystallinity of the products seems to be enhanced. The PL spectra in Figure 3b show that the intensities of exciton emission peaks at 436 nm and Mn^2+^ characteristic fluorescence peak at 595 nm gradually decrease with the increase in reaction time, and the PLQY of 41% at 15 min declines to about 19% at 60 min (Figure 3c). It is probably that the crystallization time is too long, and larger particles are formed from CsPbBr_3_, which is consistent with the XRD results, resulting in the decrease in PLQY. Figure 3d shows the photographs of the Mn^2+^ doped products synthesized at different reaction times. Under 365 nm UV light irradiation, the products at 40 and 50 min emit relatively obvious orange light. As for the 15 min-product, its orange light emission is weaker, which is caused by the large contribution of exciton blue emission.

#### 3.2.3. Effect of Mn^2+^ and Pb^2+^ Feeding Ratio

**Figure 4:**
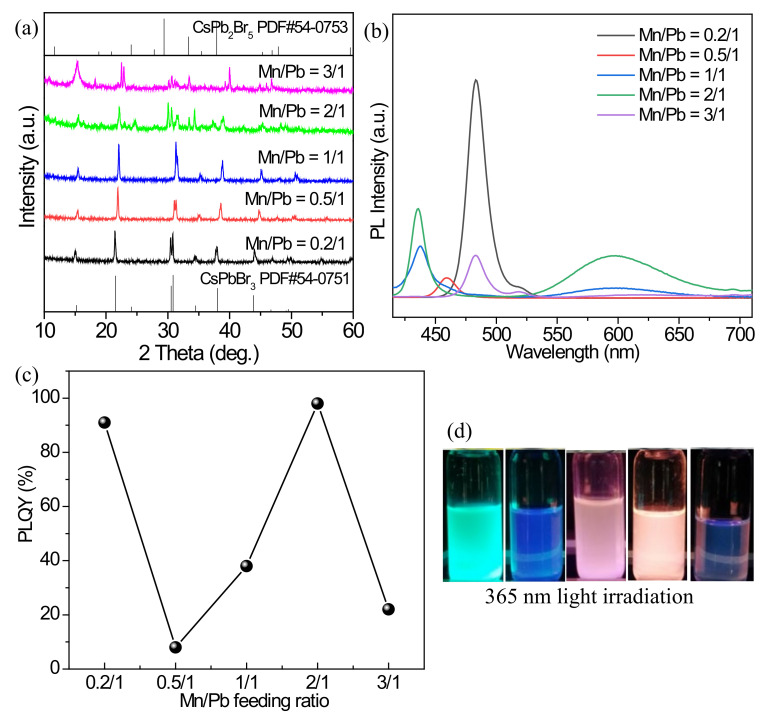
(**a**) XRD patterns, (**b**) PL emission spectra, and (**c**) PLQYs of the Mn^2+^-doped products fabricated with different Mn^2+^/Pb^2+^ feeding ratios. (**d**) Optical images of the cyclohexane solutions containing the Mn^2+^-doped products under the environments of natural and 365 nm light: from left to right, the Mn^2+^/Pb^2+^ feeding ratios are 0.2/1, 0.5/1, 1/1, 2/1, and 3/1, respectively.

Different Mn^2+^/Pb^2+^ feeding ratios also affect the luminescence properties of the Mn^2+^ doped products. Because of the use of MnCl_2_ as a Mn^2+^ source, Mn^2+^ doping also leads to the exchange of Cl^−^ and Br^−^. To ensure the experiments can be fully performed, the reaction time of 30 min is chosen, while the other conditions of a temperature of 200 °C and ODE/DGBE volume ratio of 1/1 are not changed. Figure 4a shows the XRD patterns of the products synthesized with different Mn^2+^/Pb^2+^ feeding ratios. Compared with the standard card PDF#54-0751 of CsPbBr_3_, when the Mn^2+^/Pb^2+^ feeding ratio is 0.2/1, the introduction of MnCl_2_ does not cause a significant change in the diffraction peak intensities and positions (Figure S6), and the high crystallinity is maintained. With the increase in Mn^2+^/Pb^2+^ feeding ratios, the redshifts of the diffraction peaks are present, and the crystallinity of the Mn^2+^ doped products gradually deteriorates with some impurity phases because excessive Mn^2+^ may destroy the CsPbBr_3_ crystals. The PL emission spectra of the Mn^2+^ doped products are given in Figure 4b. For the product with a feeding ratio of 0.2/1, the exciton fluorescence emission is strongest, but no obvious fluorescence emission of Mn^2+^ can be observed (Figure S7). With the increase in the Mn^2+^/Pb^2+^ feeding ratio and the introduced Cl^−^ ions, the blueshifts of the exciton emissions are clearly present, and the highest Mn^2+^ luminescence intensity appears at the ratio of 2/1. When the Mn^2+^/Pb^2+^ feeding ratio further increases to 3/1, the Mn^2+^ emission peak is largely weakened again, and the exciton emission peak even redshifts, indicating that excessive MnCl_2_ seriously affects the ion exchanges occur between Mn^2+^ and Pb^2+^ and Cl^−^ and Br^−^ in the system, and causes damage to the original perovskite structure. At the Mn^2+^/Pb^2+^ feeding ratio of 2/1, the highest PLQY of 98% is obtained (Figure 4c), so this Mn^2+^/Pb^2+^ ratio is a suitable proportion. Figure 4d shows the photographs of the products irradiated by natural and 365 nm UV light. When the Mn^2+^/Pb^2+^ feeding ratio is 0.2/1, cyan light can be emitted under UV light. As for the product with the optimum feeding ratio of 2/1, the visible orange light is emitted.

#### 3.2.4. Effect of Mn^2+^ Source

**Figure 5:**
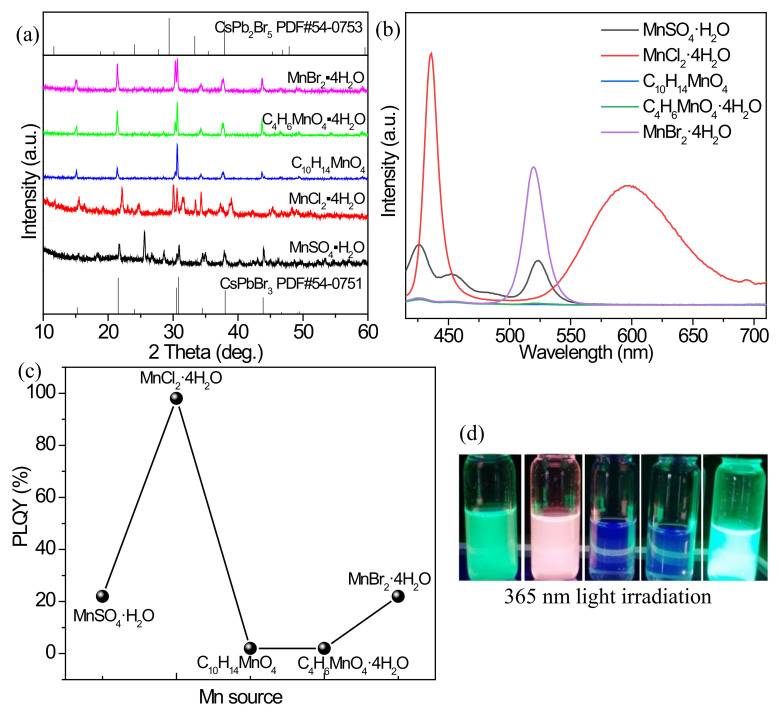
(**a**) XRD patterns, (**b**) PL emission spectra, and (**c**) PLQYs of the Mn^2+^-doped products fabricated with different Mn^2+^ precursors. (**d**) Optical images of the cyclohexane solutions containing the Mn^2+^-doped products under the environment of 365 nm light: from left to right, the Mn^2+^ precursors are MnSO_4_·H_2_O, MnCl_2_·4H_2_O, C_10_H_14_MnO_4_, C_4_H_6_MnO_4_·4H_2_O, and MnBr_2_·4H_2_O, respectively.

Different Mn^2+^ sources have different effects on the doping of Mn^2+^ and the overall luminescence of the perovskites due to the presence of different anions. Under the Mn^2+^/Pb^2+^ feeding ratio of 2/1, temperature of 200 °C, ODE/DGBE volume ratio of 1/1, and reaction time of 30 min, the products obtained by using C_10_H_14_MnO_4_, C_4_H_6_MnO_4_·4H_2_O and MnBr_2_·4H_2_O as Mn sources have high crystallinity, and their diffraction peaks correspond to the standard card PDF#54-0751 of CsPbBr_3_ (Figure 5a), whose redshift is not obvious. As for the Mn sources of MnSO_4_·H_2_O and MnCl_2_·4H_2_O, the obtained diffraction peaks are remarkably weakened, and the redshift is present, especially for the product synthesized with MnCl_2_·4H_2_O. Figure 5b shows the fluorescence spectra of the products synthesized with different Mn sources. Under the excitation of 365 nm UV light, only the product derived from MnCl_2_·4H_2_O exhibits strong exciton and Mn^2+^ emissions, and the highest PLQY of 98% is achieved (Figure 5c). In the case of C_4_H_6_MnO_4_·4H_2_O and C_10_H_14_MnO_4_, the products obtained have almost no fluorescence. With the doping sources of MnBr_2_·4H_2_O and MnSO_4_·H_2_O, the products only have exciton emissions, and their characteristic peaks of Mn^2+^ are very weak (Figure S8). Under the excitation of 365 nm UV light, the products with different Mn sources show different luminescence colors (Figure 5d), corresponding to their respective fluorescence spectra. Therefore, MnCl_2_ is the most suitable Mn source for Mn^2+^ post-doping under microwave reaction condition, while MnBr_2_ with halogen element Br cannot be successfully doped Mn^2+^ into CsPbBr_3_, indicating the ion exchange of Br^−^ and Cl^−^ is important for the doping of Mn^2+^ ions, which is consistent with the previous results [12,36]. One possible reason reported by Liu et al. is that the similar bond energy favors the doping of Mn^2+^ into CsPbCl_3_ [12]. It is noted that, with the introduction of MnCl_2_·4H_2_O, the ion exchange between Br^−^ and Cl^−^ is first occurred, resulting in the formation of Pb−Cl bond with a dissociation energy of 301 kJ/mol, which is close to that (338 kJ/mol) of Mn−Cl bond, so the doping of Mn^2+^ in CsPb(Cl/Br)_3_ can then be realized. As the Mn−Br bond in MnBr_2_, its dissociation energy (314 kJ/mol) is much stronger than that (249 kJ/mol) of Pb−Br bond, and the Mn^2+^ doped effect can be inhibited due to the extended domains of MnX_2_ over a dispersion of Mn^2+^ within the perovskite lattice [12].

#### 3.2.5. Effect of ODE/DGBE Ratio

**Figure 6:**
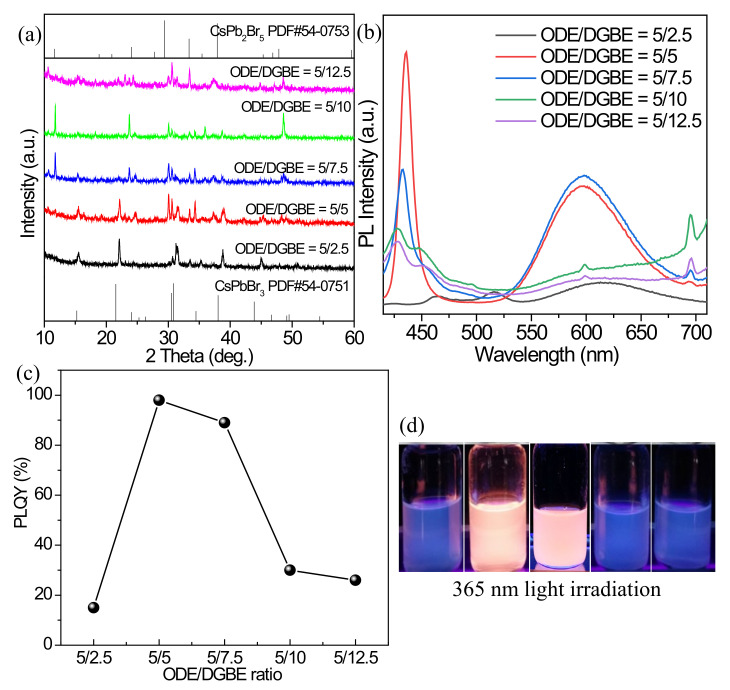
(**a**) XRD patterns, (**b**) PL emission spectra, and (**c**) PLQYs of the Mn^2+^-doped products fabricated with different ODE/DGBE ratios. (**d**) Optical images of the cyclohexane solutions containing the Mn^2+^-doped products under the environment of 365 nm light: from left to right, the ODE/DGBE ratios are 5/2.5, 5/5, 5/7.5, 5/10, and 5/12.5, respectively.

DGBE is a polar solvent that acts as a messenger of energy in the microwave reaction process. It absorbs the energy generated by microwave and then heats the solution to help the reaction be completed successfully, so the amount of DGBE also plays a very important role in the postsynthesis of the Mn^2+^ doped inorganic perovskites. The effect of the volume ratio of ODE/DGBE on the products is investigated under the conditions of MnCl_2_·4H_2_O source, Mn^2+^/Pb^2+^ feeding ratio of 2/1, temperature of 200 °C, and reaction time of 30 min. When the ODE/DGBE ratios are changed from 5/2.5 to 5/5, the redshifted diffraction peaks of the products are present compared with the standard card PDF#54-0751 of CsPbBr_3_ (Figure 6a). At the ODE/DGBE ratios of 5/7.5 and 5/10, some diffraction peaks attributing to CsPb_2_Br_5_ also appear in the products. As the ODE/DGBE ratio further increases to 5/12.5, the intensity of the diffraction peaks largely decreases, indicating the low crystallinity of perovskite phases. The PL emission spectra of the products synthesized with different ODE/DGBE volume ratios are given in Figure 6b. At the low ODE/DGBE ratio of 5/2.5, both the exciton and Mn^2+^ characteristic peaks are weak. When the high ODE/DGBE ratios of 5/10 and 5/12.5 are applied, there are no obvious characteristic emission peaks of Mn^2+^. The strong Mn^2+^ characteristic peaks are present in the products synthesized with the ODE/DGBE ratios of 5/5 and 5/7.5, and although there is a slight decrease in the intensity of the former, its PLQY (98%) is stronger than that (89%) of the latter (Figure 6c). Therefore, the ODE/DGBE ratio of 5/5 is the most appropriate value for the postsynthesis of the Mn^2+^-doped product, which emits bright orange light under the excitation of 365 nm UV light (Figure 6d).

### 3.3. Optical Properties before and after Mn^2+^ Doping

**Figure 7:**
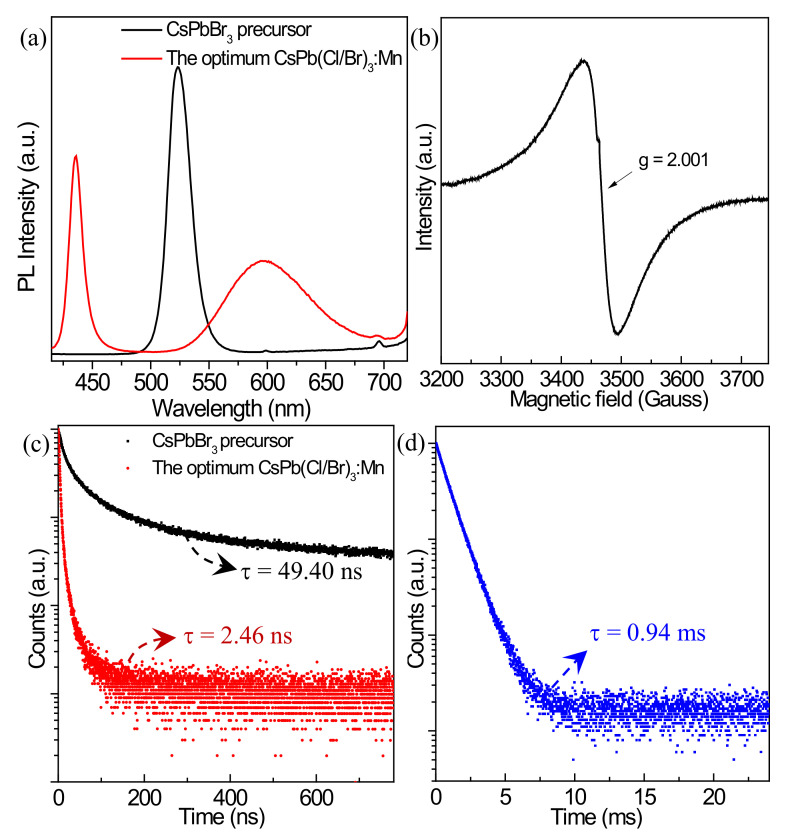
(**a**) PL emission spectra of the CsPbBr_3_ precursor and the optimum CsPb(Cl/Br)_3_:Mn product. (**b**) EPR spectrum of the optimum CsPb(Cl/Br)_3_:Mn product. (**c**) PL decay curves of the CsPbBr_3_ precursor and CsPb(Cl/Br)_3_:Mn detected at 523 and 437 nm, respectively, with 370 nm excitation. (**d**) PL decay curve of CsPb(Cl/Br)_3_:Mn detected at 597 nm with 370 nm excitation.

According to the above results, the optimum Mn^2+^-doped product is synthesized by using the MnCl_2_·4H_2_O source and the conditions of a temperature of 200 °C, reaction time of 30 min, Mn^2+^/Pb^2+^ feeding ratio of 2/1, and ODE/DGBE volume ratio of 5/5, which is marked as CsPb(Cl/Br)_3_:Mn (Figure S9). As shown in Figure 7a, through Mn^2+^ doping, the exciton fluorescence emission peak of the CsPbBr_3_ precursor blueshifts from 523 to 437 nm of CsPb(Cl/Br)_3_:Mn, and although the exciton peak decreases, a large Mn^2+^ characteristic emission peak at 597 nm is obtained, suggesting the Mn^2+^ ions have been doped into the CsPbBr_3_ crystals. The Mn^2+^ doping effect can also be demonstrated by the EPR spectrum in Figure 7b, where the single peak with a g value of 2.001 means the Mn^2+^ ions are indeed present in the crystals [37,38,39], and the lack of six hyperfine splitting profile is caused by the strong exchange interaction between Mn-pairs introduced from the high feeding dosage of Mn^2+^/Pb^2+^. The UV-vis absorption spectra of the products before and after Mn^2+^ doping are given in Figure S10, and the initial first excitonic absorption peak at 515 nm is blueshifted to about 436 nm. The PL decay curves of the exciton peaks are given in Figure 7c, and the average PL lifetime (2.46 ns) of CsPb(Cl/Br)_3_:Mn is much lower than that (49.40 ns) of the CsPbBr_3_ precursor, indicating there is an efficient energy transfer process from exciton to Mn^2+^, leading to the generation of the Mn^2+^ emission peak with an average PL lifetime of 0.94 ms (Figure 7d), which is originated from the ^4^T_1_→^6^A_1_ transitions of the Mn^2+^ ions [12,32]. The PLQY of the CsPbBr_3_ precursor is calculated to be 93%, and after Mn^2+^ doping, the total PLQY of the optimum CsPb(Cl/Br)_3_:Mn improves to 98%. Therefore, Mn^2+^ doping provides the enhanced PL property for the inorganic perovskites.

### 3.4. Morphology Evolution before and after Mn^2+^ Doping

**Figure 8:**
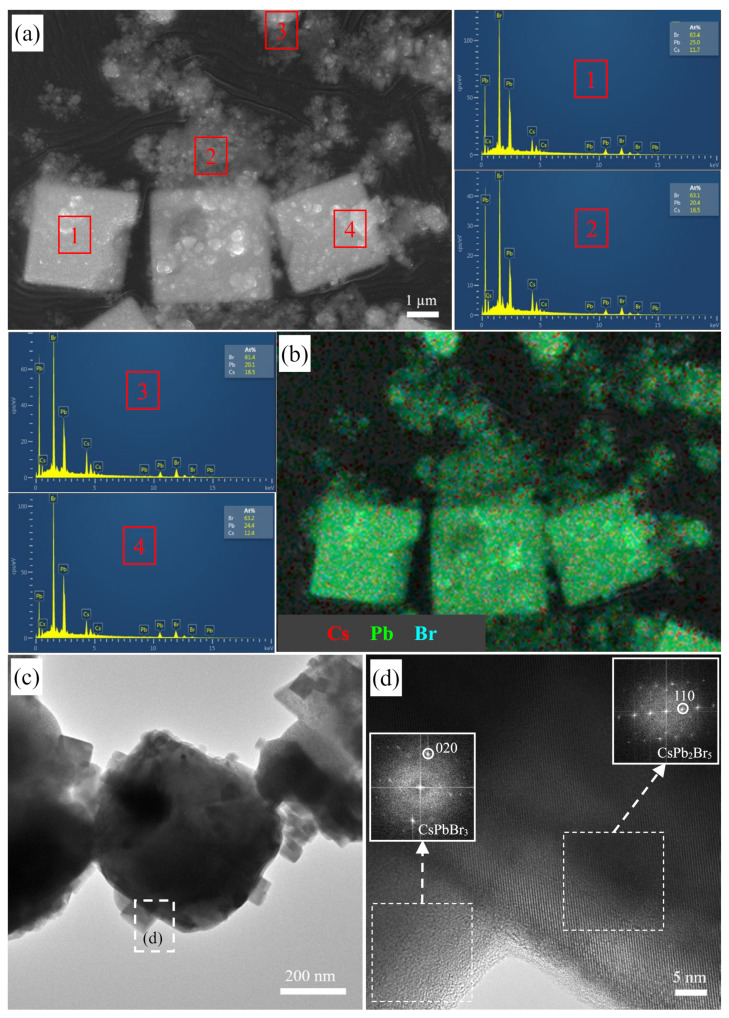
(**a**) SEM image of the CsPbBr_3_ precursor and its corresponding EDS spectra of the points 1, 2, 3, and 4. (**b**) The elemental overlay of Cs, Pb, and Br for the CsPbBr_3_ precursor. (**c**) TEM and (**d**) HRTEM images of the CsPbBr_3_ precursor; insets: FFT patterns.

The morphology of the CsPbBr_3_ precursor is analyzed by SEM. As shown in Figure 8a, there are mainly two kinds of particles: small particle aggregates and microcubic particles with an average size of about 3.4 ± 0.4 μm. To distinguish them, the EDS spectra of points 1, 2, 3, and 4 selected from Figure 8a are tested, and the Cs:Pb:Br molar ratios of the small particle aggregates are close to 1:1:3 (Table S1), while those of the microcubic particles are close to 1:2:5. The EDS mapping results of Figure 8a are provided in Figure 8b and Figure S11, and the Cs signals (red color) in the small particle aggregates are distributed more densely compared with those in the large square particles. According to the crystal phases contained in the CsPbBr_3_ precursor, it can be indicated that the microcubic particles are attributed to CsPb_2_Br_5_, and the small particle aggregates mainly consist of CsPbBr_3_ NCs. Figure 8c shows the TEM image of the CsPbBr_3_ precursor, and the microcubic aggregate particles and cubic NCs can be observed. The corresponding high-resolution TEM (HRTEM) image is given in Figure 8d, and the (020) planes of CsPbBr_3_ in the cubic NC and the (110) planes of CsPb_2_Br_5_ in the microcubic particle are present in the fast Fourier transform (FFT) patterns (insets of Figure 8d). In combination with the SEM results, it can be inferred that the surface of the CsPb_2_Br_5_ microcubic particles is deposited with many well-separated CsPbBr_3_ NCs, which ensure the high luminous performance of the CsPbBr_3_ precursor.

**Figure 9:**
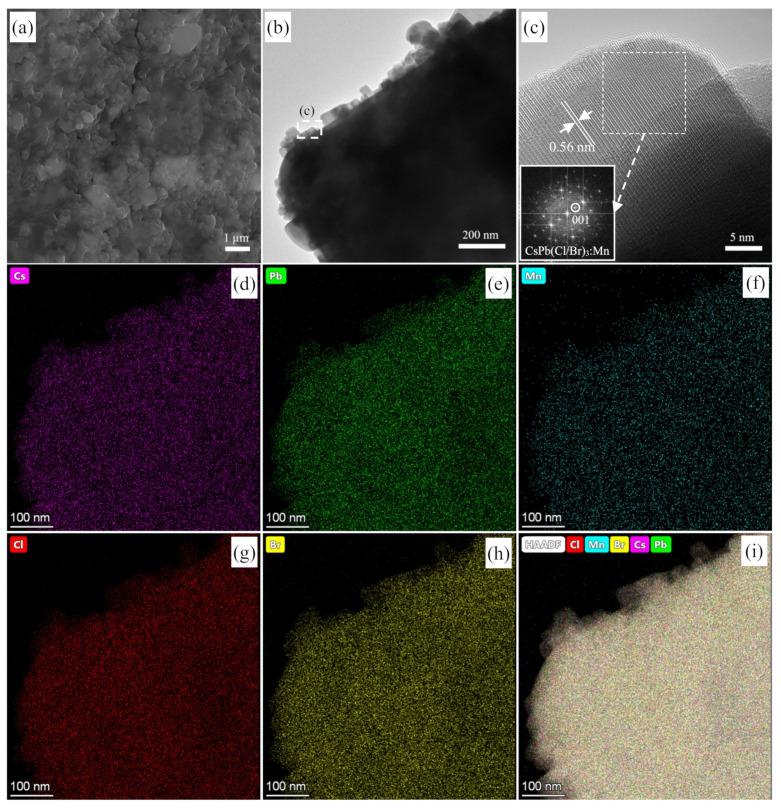
(**a**) SEM, (**b**) TEM and (**c**) HRTEM images of the optimum CsPb(Cl/Br)_3_:Mn product; inset: FFT pattern. The elemental maps of (**d**) Cs, (**e**) Pb, (**f**) Mn, (**g**) Cl, (**h**) Br, and (**i**) their overlay with the HAADF-STEM image of the optimum CsPb(Cl/Br)_3_:Mn product.

The SEM morphology of the optimum CsPb(Cl/Br)_3_:Mn product is shown in Figure 9a, where the aggregate particles consisting of nanoparticles are present. From the TEM image of CsPb(Cl/Br)_3_:Mn (Figure S12), it can be found that the microcubic particles with an average size of about 3.2 ± 0.3 μm are also present, but they are not attributed to CsPb_2_Br_5_, because it is disappeared after Mn^2+^ doping. Importantly, there are many cubic NCs on the outmost surface of microcubic particles (Figure 9b). To confirm the structure of these NCs, their HRTEM image is tested and shown in Figure 9c. The lattice fringe spacing of the cubic NC is measured to be 0.56 nm, which consists of the (001) planes (FFT pattern of Figure 9c) of the partial Cl^−^-substituted CsPb(Cl/Br)_3_:Mn. The high-angle annular dark field scanning TEM (HAADF-STEM) image of CsPb(Cl/Br)_3_:Mn is provided in Figure S13, and the STEM-EDS maps of Cs (Figure 9d), Pb (Figure 9e), Mn (Figure 9f), Cl (Figure 9g), and Br (Figure 9h) indicate that they are uniformly distributed throughout the microcubic particle (Figure 9i), including the outmost surface NCs. Therefore, the microcubic particles and their surface NCs are all ascribed to the CsPb(Cl/Br)_3_:Mn phase, wherein the microcubic morphology is probably inherited from the large CsPb_2_Br_5_ particles, while the presence of the surface well-separated CsPb(Cl/Br)_3_:Mn NCs is the guarantee of the high PLQY and excellent color-tunable luminescence property of the Mn^2+^ doped product since the contribution from their microcrystals is very limited. The detected molar ratio of Cs:Pb:Br:Cl:Mn is 1:0.99:1.65:3.8:1.3 (Table S1), meaning a certain amount of Mn^2+^ has been doped into the CsPbBr_3_ precursor to form CsPb(Cl/Br)_3_:Mn NCs, which possess the obvious Mn^2+^-related luminescence.

### 3.5. XPS Analysis before and after Mn^2+^ Doping

**Figure 10:**
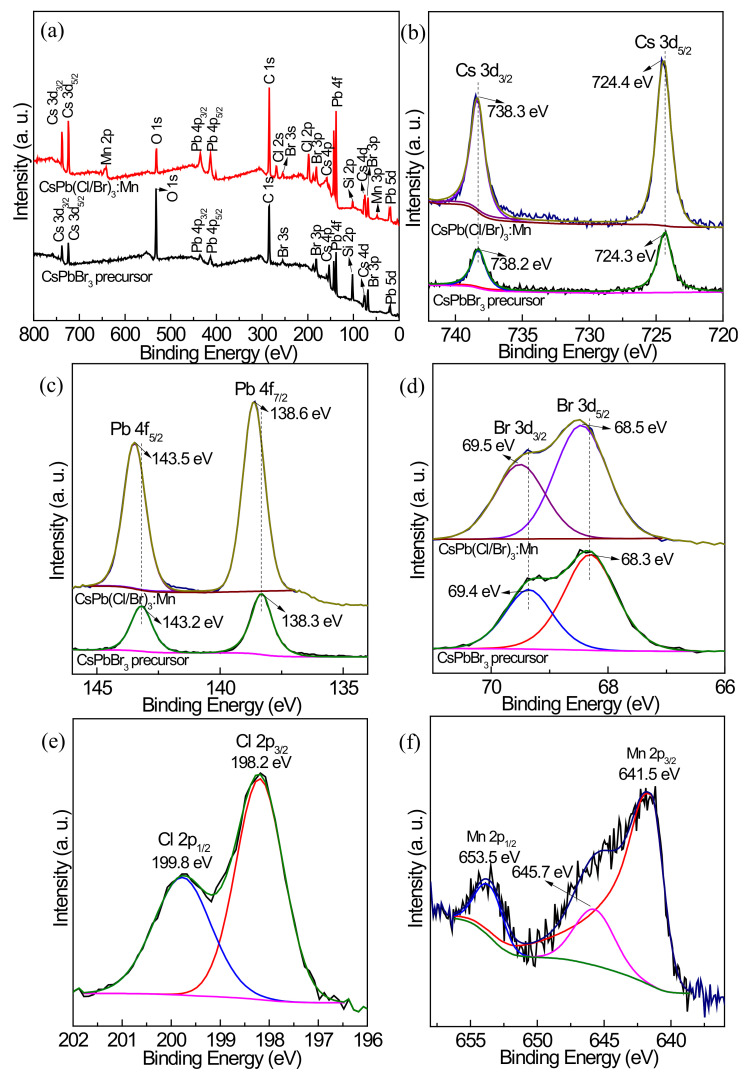
(**a**) Survey scan XPS spectra of the CsPbBr_3_ precursor and the optimum CsPb(Cl/Br)_3_:Mn product and their high-resolution XPS spectra of (**b**) Cs 3d, (**c**) Pb 4f, and (**d**) Br 3d. High-resolution XPS spectra of the optimum CsPb(Cl/Br)_3_:Mn product: (**e**) Cl 2p and (**f**) Mn 2p.

The change of surface composition and chemical states before and after Mn^2+^ doping is investigated by XPS. As shown in the survey scan XPS spectrum of the CsPbBr_3_ precursor (Figure 10a), the elements Cs, Pb, Br, C, O, and Si are detected, where the C and O signals are mainly generated from the residual surface ligands, such as OA, OLA, ODE, and DGBE, and the Si signal is produced by the used glass substrate. After Mn^2+^ doping treatment with MnCl_2_·4H_2_O, the additional spectroscopic signatures of Mn and Cl appear in the optimum CsPb(Cl/Br)_3_:Mn product. The high-resolution Cs 3d spectra are shown in Figure 10b, where two peaks corresponding to Cs 3d_3/2_ and Cd 3d_5/2_ are recorded. The binding energies of the CsPbBr_3_ precursor are at 738.2 and 724.3 eV, which respectively shift to higher values of 738.3 and 724.4 eV for CsPb(Cl/Br)_3_:Mn. For the Pb 4f spectra (Figure 10c), two obvious peaks at 143.2 and 138.3 eV for the CsPbBr_3_ precursor and those at 143.5 and 138.6 eV for CsPb(Cl/Br)_3_:Mn are detected. The Br 3d spectra can be resolved into two peaks at 69.4 and 68.3 eV for the CsPbBr_3_ precursor and those at 69.5 and 68.5 eV for CsPb(Cl/Br)_3_:Mn (Figure 10d). As a result, after Mn^2+^ doping, the Pb 4f and Br 3d peaks also shift to higher binding energies, which should be caused by the modified chemical environment and changed electron density [36]. The Cl 2p spectrum of CsPb(Cl/Br)_3_:Mn is given in Figure 10e, and the two peaks at 199.8 and 198.2 eV are ascribed to Cl 2p_1/2_ and Cl 2p_3/2_, respectively. Figure 10f shows the Mn 2p spectrum of CsPb(Cl/Br)_3_:Mn, and three peaks can be fit. The peaks at 653.5 and 641.5 eV are attributed to Mn 2p_1/2_ and Mn 2p_3/2_, respectively, and the peak at 645.7 eV is assigned to the Mn 2p core level [36], meaning Mn^2+^ ions have been successfully doped into the crystal lattice of CsPbBr_3_. According to the XPS results, the molar ratio of Cs:Pb:Br for the CsPbBr_3_ precursor is calculated to be 1:1.7:13.2 (Table S1), indicating its surface is enriched with Br element. After Mn^2+^ doping, the Cs:Pb:Br:Cl:Mn molar ratio is calculated to be 1:2.5:8.2:7.1:1.4 for CsPb(Cl/Br)_3_:Mn, and its surface is also enriched with the halogen elements. These excess halogen signals are different from the results obtained from SEM-EDS and STEM-EDS (Table S1), which may be caused by the excess introduced PbBr_2_, MnCl_2_, and organic capping ligands in the synthesis process. However, the XPS results have proved that there are a certain number of Mn^2+^ and Cl^−^ ions located on the outmost surface of CsPb(Cl/Br)_3_:Mn NCs.

### 3.6. Stability before and after Mn^2+^ Doping

**Figure 11:**
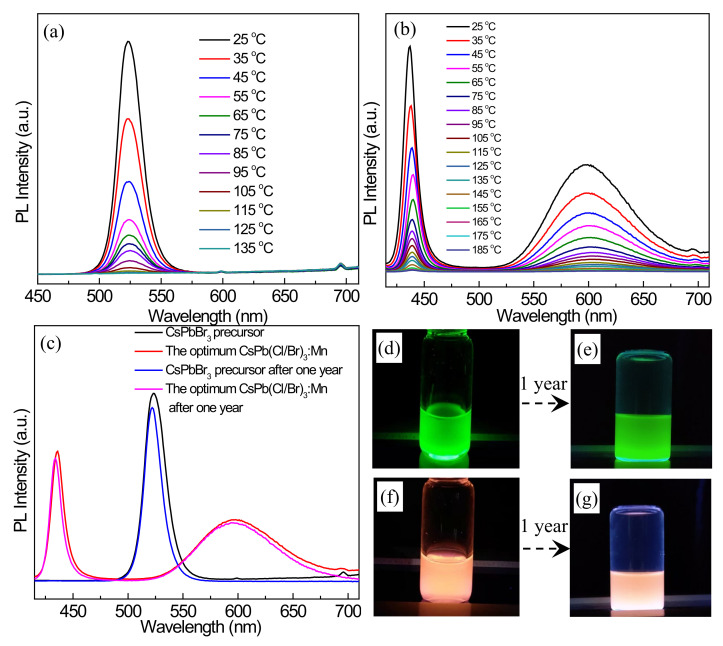
PL emission spectra of (**a**) the CsPbBr_3_ precursor and (**b**) the optimum CsPb(Cl/Br)_3_:Mn product as the treatment temperatures increase. (**c**) PL emission spectra of the CsPbBr_3_ precursor and the optimum CsPb(Cl/Br)_3_:Mn product before and after storing in air for one year. Optical images of the cyclohexane solutions containing (**d**,**e**) the CsPbBr_3_ precursor and (**f**,**g**) the optimum CsPb(Cl/Br)_3_:Mn product under the environment of 365 nm light before and after storing in air for one year.

The thermal stabilities of the perovskite materials before and after Mn^2+^ doping are first investigated by the temperature-dependent PL experiments. The PL spectra of the CsPbBr_3_ precursor at temperatures varying from 25 to 135 °C are shown in Figure 11a. It can be found that, with the increase in temperature, the PL peak at 523 nm decreases significantly. When the temperature reaches 135 °C, almost no PL emission is found for the CsPbBr_3_ precursor. Figure 11b shows the PL spectra of the optimum CsPb(Cl/Br)_3_:Mn product tested at the temperatures change from 25 to 185 °C. With the increase in temperature, the exciton emission peak at 437 nm and the Mn^2+^ emission peak at 597 nm decreases synchronously. At 135 °C, two distinct emission peaks could still be seen, and the remnant PL of the exciton emission peak is about 5.04%, which is higher than that (0.93%) of the CsPbBr_3_ precursor (Figure S14a). The corresponding PL positions of the exciton emissions are provided in Figure S14b, and the change range (437 to 439.5 nm) of CsPb(Cl/Br)_3_:Mn is smaller than that (523 to 527 nm) of the CsPbBr_3_ precursor. All these indicate that CsPb(Cl/Br)_3_:Mn has higher thermal stability compared with that of the CsPbBr_3_ precursor.

To further compare the stability of the products before and after Mn^2+^ doping, their PL emission spectra after storing in air for one year are tested. As shown in Figure 11c, the PL decrease in the CsPbBr_3_ precursor and CsPb(Cl/Br)_3_:Mn is not obvious, and high PLQYs of 86% and 93% are respectively maintained after one year. Figure 11d–g shows the optical images of them, and the luminescence color of the CsPbBr_3_ precursor is changed from bright green (Figure 11d) to bright orange (Figure 11f) for CsPb(Cl/Br)_3_:Mn, which almost maintain the same even after one year. The maintenance of the PLQYs and the color-tunable luminescence properties suggests the high stability of the products fabricated by using microwave heating.

**Figure 12:**
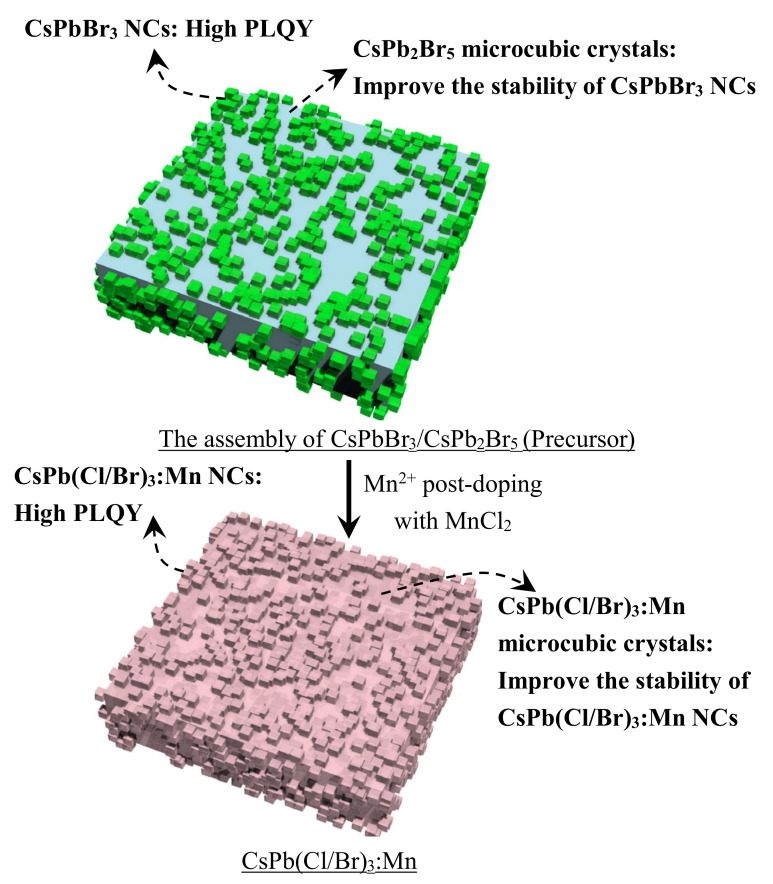
Schematics of PL and stability enhancement mechanism of the CsPbBr_3_ precursor and the optimum CsPb(Cl/Br)_3_:Mn product.

On the basis of the above morphology and composition analysis (Figure 8 and Figure 10), there are two crystal phases of CsPbBr_3_ and CsPb_2_Br_5_ contained in the CsPbBr_3_ precursor, where the CsPbBr_3_ NCs are dispersed on the surface of the CsPb_2_Br_5_ microcubic crystals with sizes of about 3.4 ± 0.4 μm. These well-separated CsPbBr_3_ NCs are responsible for the strong 523 nm PL emission of the precursor (Figure 7 and Figure 12), while the CsPb_2_Br_5_ microcubic crystals can be taken as the substrates to embed CsPbBr_3_ NCs to boost their stability. After the post-doping of Mn^2+^ ions, the CsPb_2_Br_5_ phase disappeared, and the redshifted diffraction peaks of CsPb(Cl/Br)_3_:Mn appeared (Figure 4a, Figure 5a, and Figure 6a). In terms of the morphology and composition of CsPb(Cl/Br)_3_:Mn (Figure 9, Figure 10, and Figure S12), there are also microcubic crystals with sizes of about 3.2 ± 0.3 μm and NCs. These microcubic crystals are very likely formed from the CsPb_2_Br_5_ phases after their ion exchange of Br^−^ by Cl^−^ and the doping of Mn^2+^ ions. Meanwhile, the CsPb(Cl/Br)_3_:Mn NCs can also be produced from the CsPbBr_3_ NCs and maintain their positions to be uniformly and tightly dispersed on the surface of the CsPb(Cl/Br)_3_:Mn microcubic crystals (Figure 12). Furthermore, the surface organic capping ligands of the CsPbBr_3_, CsPb_2_Br_5_, and CsPb(Cl/Br)_3_:Mn are almost the same (Figure S15) [40,41]. Therefore, the physical and chemical adsorptions may be present for the interaction between the NCs and the microcubic crystals, which greatly enhance the stability of the system. It is worth noting that although the microsized perovskite crystals are formed from this microwave-assisted method, the NCs can be generated and embedded on the surface of the microcubic crystals, leading to the formation of excellent color-tunable luminescent perovskite materials with high stability.

## 4. Conclusions

The assembly of CsPbBr_3_ NCs deposited on CsPb_2_Br_5_ microcubic crystals is initially prepared by a microwave method, where the feed mole ratio of CsCO_3_/PbBr_2_, temperature, and volume ratio of ODE/DGBE are investigated, and the sample with best luminescent performance is used as the precursor. The Mn^2+^ post-doping experiments are based on the precursor. The effects of reaction temperatures, times, Mn^2+^/Pb^2+^ feeding ratios, Mn^2+^ sources, and ODE/DGBE ratios are performed, and the optimum CsPb(Cl/Br)_3_:Mn product is achieved. With the introduction of MnCl_2_·4H_2_O, the exciton fluorescence emission peak of the CsPbBr_3_ precursor is blueshifted from 523 to 437 nm of CsPb(Cl/Br)_3_:Mn due to the partial substitution of Br^−^ by Cl^−^, and a new Mn^2+^ characteristic emission peak at 597 nm is produced. For the CsPbBr_3_ precursor, the high PLQY is generated from the well-separated CsPbBr_3_ NCs on the CsPb_2_Br_5_ microcubic crystals, which also improve the stability of CsPbBr_3_ NCs. After Mn^2+^-doping treatment, the microcubic crystal shape of CsPb_2_Br_5_ shape is kept to form CsPb(Cl/Br)_3_:Mn microcrystals, whose surface is also well dispersed and embedded with CsPb(Cl/Br)_3_:Mn NCs to gain high PLQY and stability.

## Data Availability

The data is available on reasonable request from the corresponding author.

## Supplementary Materials

The following supporting information can be downloaded at: https://www.mdpi.com/article/10.3390/nano12152535/s1. Figures S1–S4, S7, S8, and S9: PL emission spectra, Figures S5 and S6: XRD patterns, Figure S10: UV-vis absorption spectra, Table S1: Element contents, Figure S11: Elemental maps, Figure S12: TEM image, Figure S13: HAADF-STEM image, and Figure S14: The changes of PL peak intensities and positions of the products. References [40,41] are cited in the supplementary materials.
