# Supplementary material for: Systematic Microwave-Assisted Postsynthesis of Mn-Doped Cesium Lead Halide Perovskites with Improved Color-Tunable Luminescence and Stability
Source: Nanomaterials (Basel). 2022 Jul 23;12(15):2535. doi: 10.3390/nano12152535 (PMC9351682; doi:10.3390/nano12152535)
Supplement: Supplementary file 1 [file nanomaterials-12-02535-s001.zip › nanomaterials-1724635-supplementary.pdf]

nanomaterials

Supplementary Material

Systematic Microwave-Assisted Postsynthesis of Mn-Doped Cesium Lead Halide Perovskites with Improved Color-Tunable Luminescence and Stability

Yaheng Zhang [1], Chao Fan [1], Jianghong Tang [1], Gaoming Huang [1], Xinfa Qiang [2], Yu Fu [1], Wenjuan Zhou [1], Juan Wu [1] and Shouqiang Huang [1,*]

[1] Jiangsu Key Laboratory of E-waste Recycling, School of Chemistry and Environmental Engineering, Jiangsu University of Technology, Changzhou 213001, China; zhangyaheng@jsut.edu.cn (Y.Z.); chaofan19930703@163.com (C.F.); tjh01@jsut.edu.cn (J.T.); hanggaoming1122@163.com (G.H.); fuyu@jsut.edu.cn (Y.F.); 2019560060@jsut.edu.cn (W.Z.); jintanwujuan@163.com (J.W.)
[2] Jiangsu Key Laboratory of Advanced Structural Materials and Application Technology, Nanjing Institute of Technology, Nanjing 211167, China; qiangxinfa@163.com
* Correspondence: hshouqiang@126.com

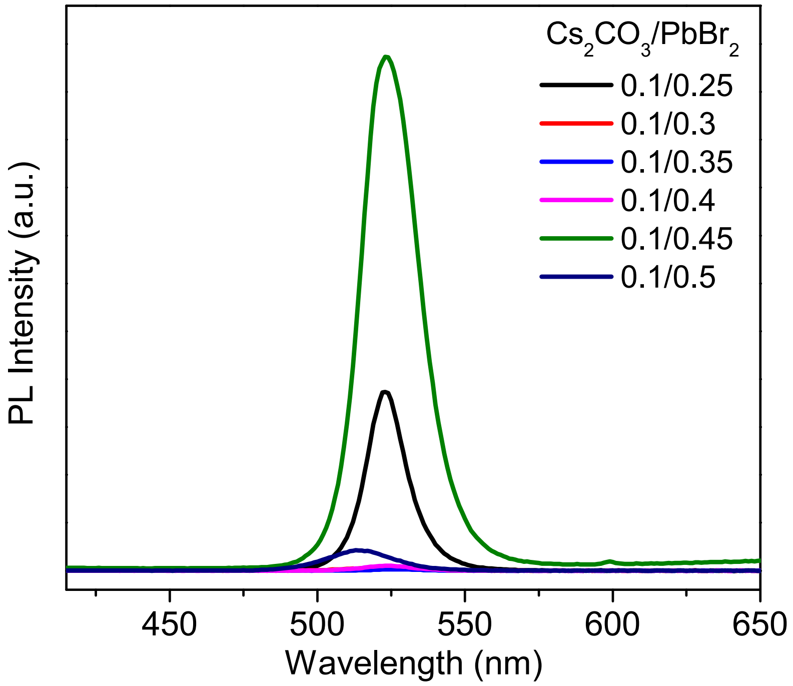

Figure S1. PL emission spectra of the products prepared at different feed mole ratios of $Cs_2CO_3/PbBr_2$.

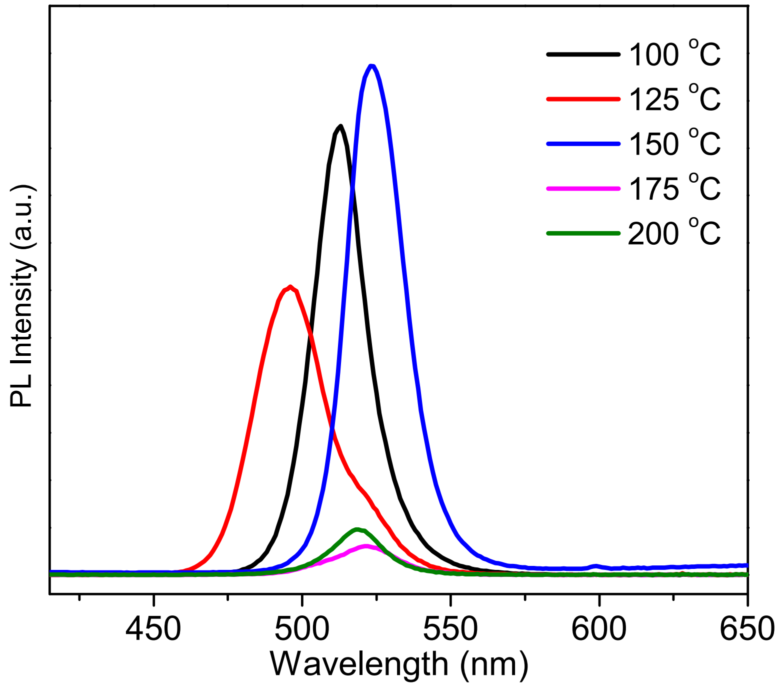

Figure S2. PL emission spectra of the products prepared at different reaction temperatures.

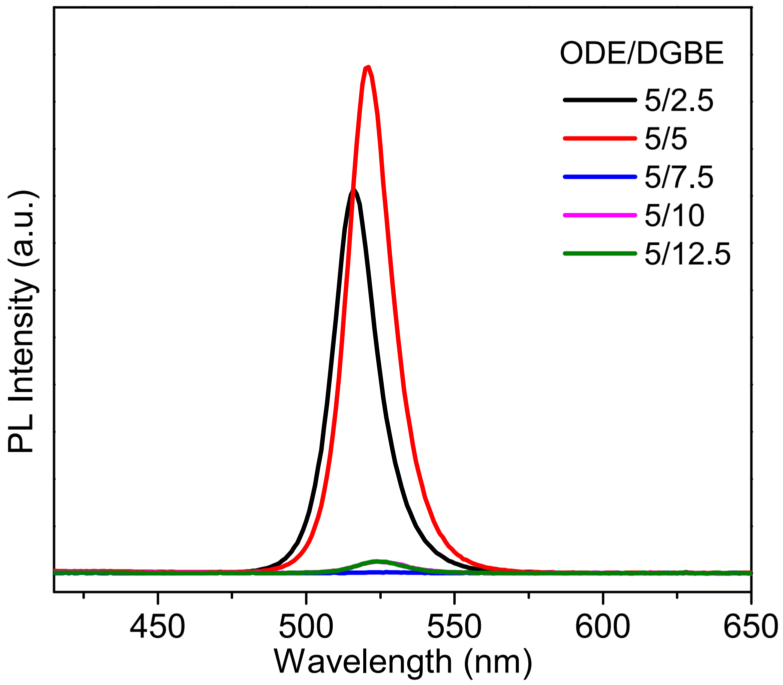

Figure S3. PL emission spectra of the products prepared at different volume ratios of ODE/DGBE.

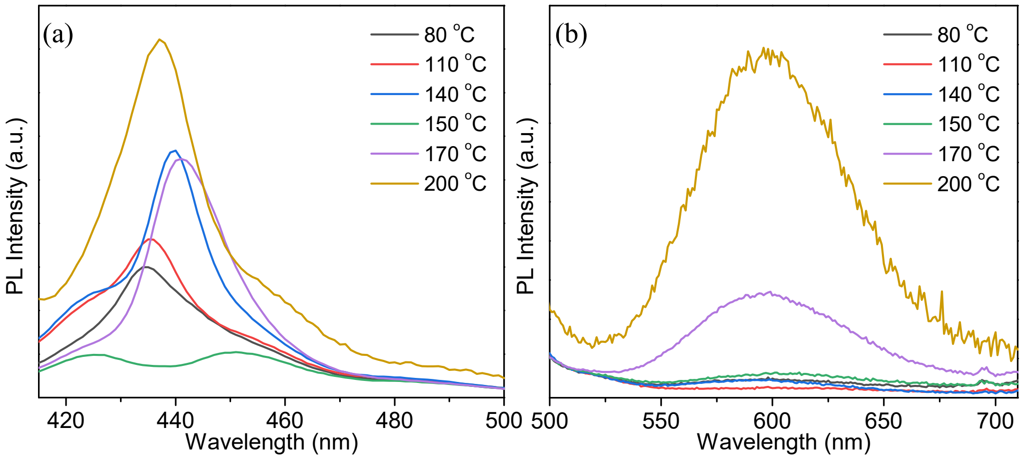

Figure S4. The enlarged PL emission spectra of the pristine $CsPbBr_3$ doped with Mn^{2+} ions fabricated at different reaction temperatures: (a) 380 ~ 500 nm and (b) 500 ~ 710 nm.

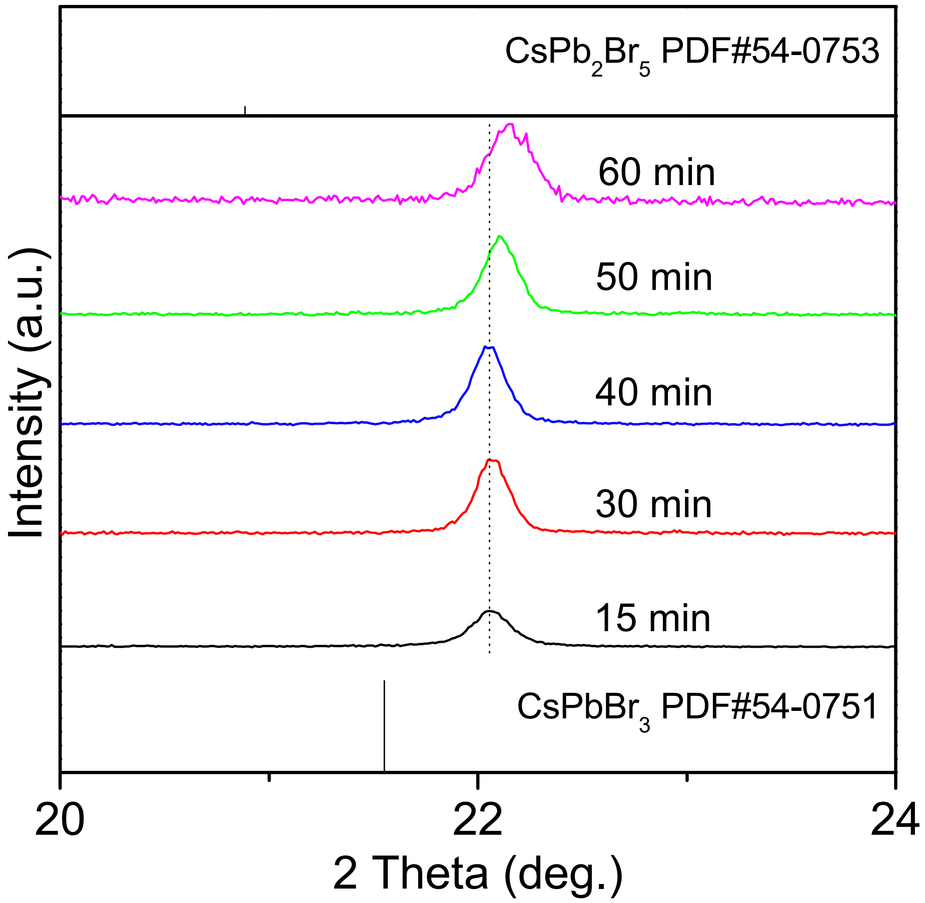

Figure S5. The enlarged XRD patterns of the pristine $CsPbBr_3$ doped with Mn^{2+} ions fabricated with different reaction times in the range of 2θ from 20° to 24°.

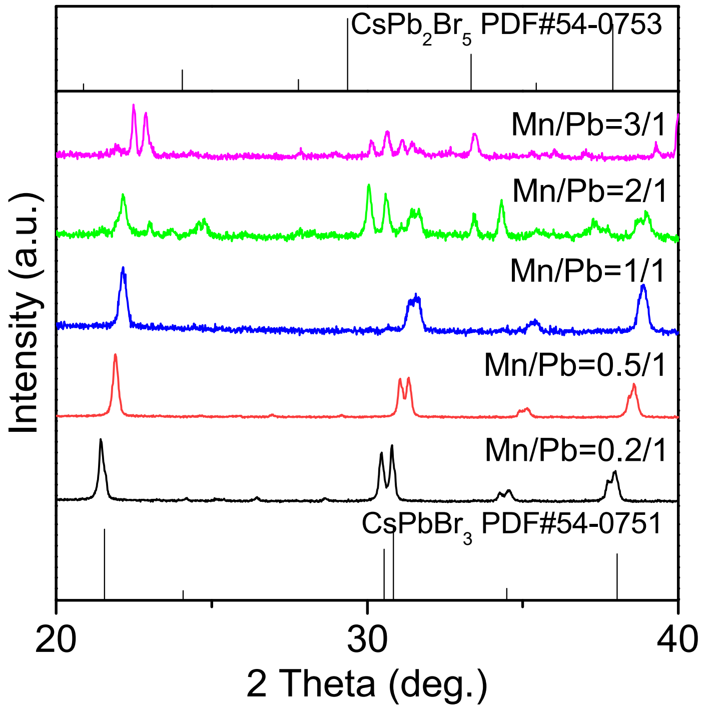

Figure S6. The enlarged XRD patterns of the pristine $CsPbBr_3$ doped with Mn^{2+} ions fabricated with different Mn^{2+}/Pb^{2+} feeding ratios in the range of 2θ from 20º to 40º.

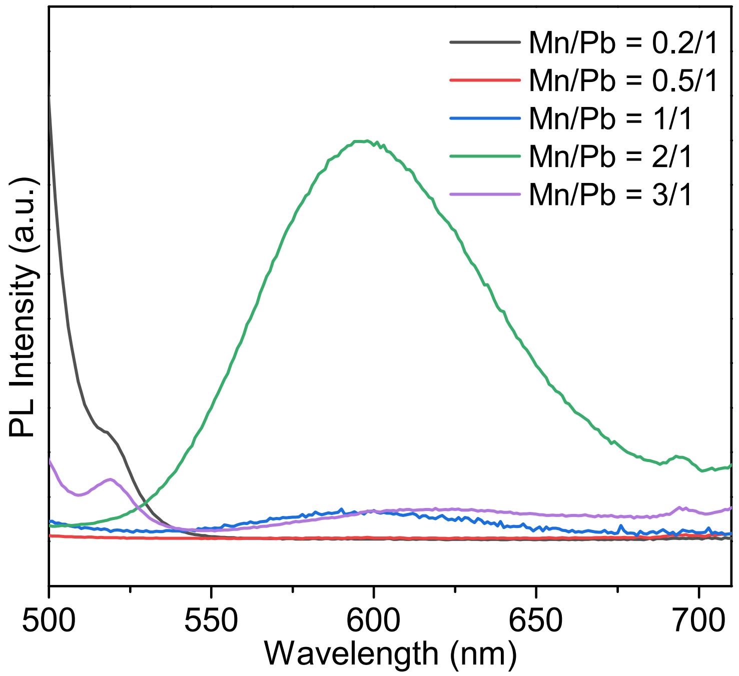

Figure S7. The enlarged PL emission spectra of the pristine $CsPbBr_3$ doped with Mn^{2+} ions fabricated at different Mn^{2+} and Pb^{2+} feeding ratios.

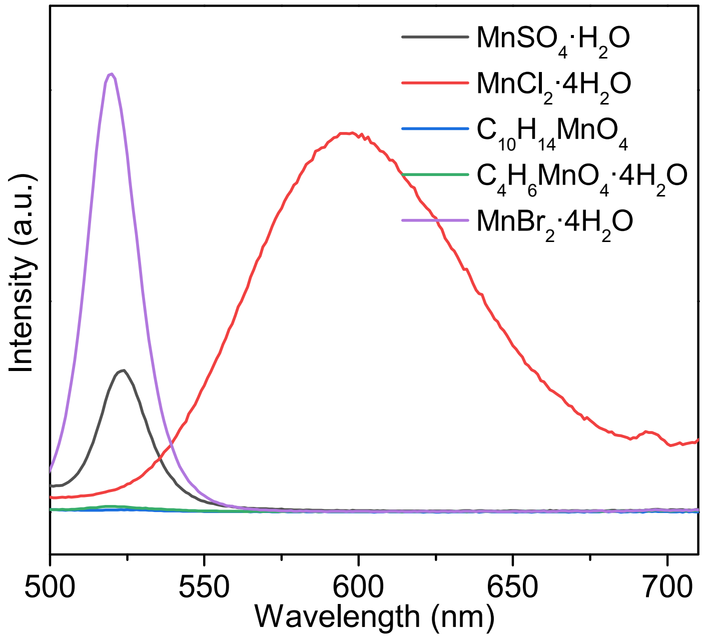

Figure S8. The enlarged PL emission spectra of the pristine $CsPbBr_3$ doped with Mn^{2+} ions fabricated fabricated with different Mn^{2+} sources.

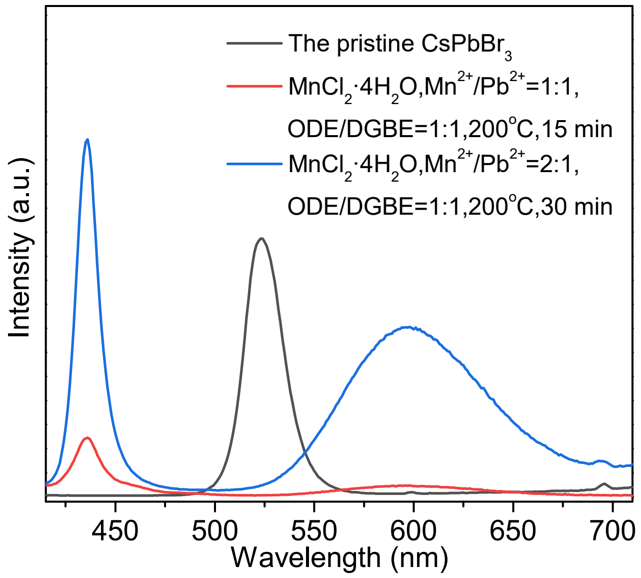

Figure S9. PL emission spectra of the pristine $CsPbBr_3$ and the doped products selected from the comparison of reaction time and the Mn^{2+}/Pb^{2+} feeding ratio. The product synthesized under the conditions of $MnCl_2·4H_2O$ source, Mn^{2+}/Pb^{2+} = 2/1, ODE/DGBE = 5/5, 200 °C and reaction time of 30 min has the strongest PL emission, which is marked as the optimum $CsPb(Cl/Br)_3$:Mn product.

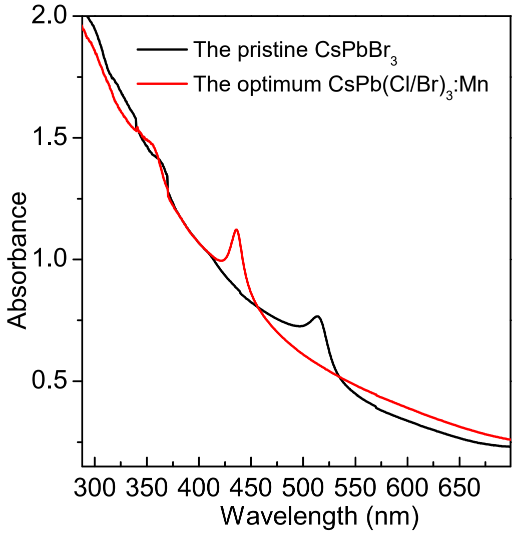

Figure S10. UV-vis absorption spectra of the pristine $CsPbBr_3$ and the optimum $CsPb(Cl/Br)_3$:Mn product.

Table S1. Element contents of the pristine $CsPbBr_3$ and the optimum $CsPb(Cl/Br)_3$:Mn product obtained from SEM-EDS, STEM-EDS, and XPS.

**Elements**			**Atomic%**				
			**Cs**	**Pb**	**Br**	**Cl**	**Mn**
**The pristine $CsPbBr_3$**	SEM-EDS	Point 1	11.7	25.0	63.4	—	—
			Cs: Pb: Br = 1: 2.1: 5.4				
		Point 2	16.5	20.4	63.1	—	—
			Cs: Pb: Br = 1: 1.2: 3.8				
		Point 3	18.5	20.1	61.4	—	—
			Cs: Pb: Br = 1: 1.1: 3.3				
		Point 4	12.4	24.4	63.2	—	—
			Cs: Pb: Br = 1: 2.0: 5.1				
		**C**	**Cs**	**Pb**	**Br**	**Cl**	**Mn**
**The pristine $CsPbBr_3$**	XPS	89.96	0.63	1.09	8.32	—	—
		Cs: Pb: Br = 1: 1.7: 13.2					
		**C**	**Cs**	**Pb**	**Br**	**Cl**	**Mn**
**$CsPb(Cl/Br)_3$:Mn**	STEM-EDS	—	11.5	11.4	19.0	43.7	14.4
		Cs: Pb: Br: Cl: Mn = 1: 0.99: 1.65: 3.8: 1.3					
	XPS	76.38	1.17	2.87	9.61	8.35	1.62
		Cs: Pb: Br: Cl: Mn = 1: 2.5: 8.2: 7.1: 1.4					

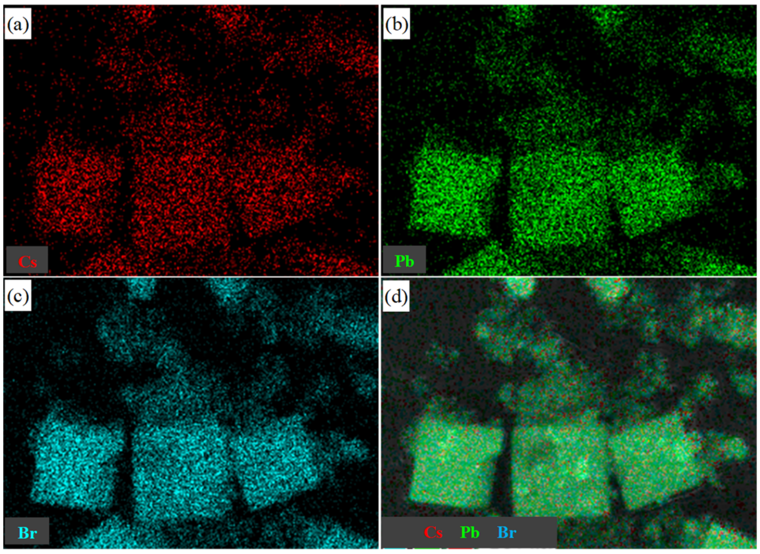

Figure S11. The elemental maps of the pristine $CsPbBr_3$: (a) Cs, (b) Pb, (c) Br, and (d) their overlay.

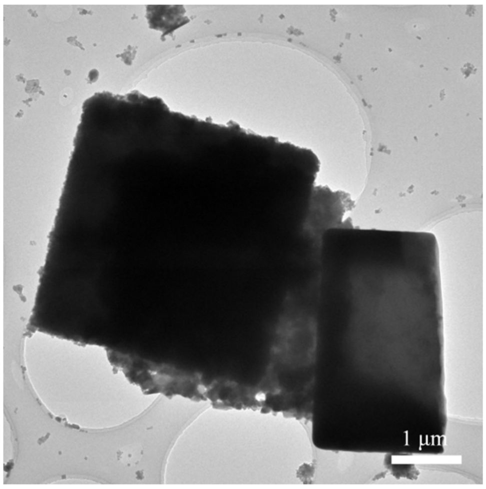

Figure S12. TEM image of the optimum $CsPb(Cl/Br)_3$:Mn product.

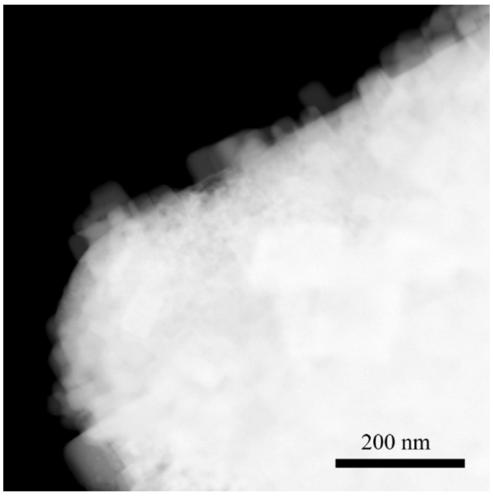

Figure S13. HAADF-STEM image of the optimum $CsPb(Cl/Br)_3$:Mn product.

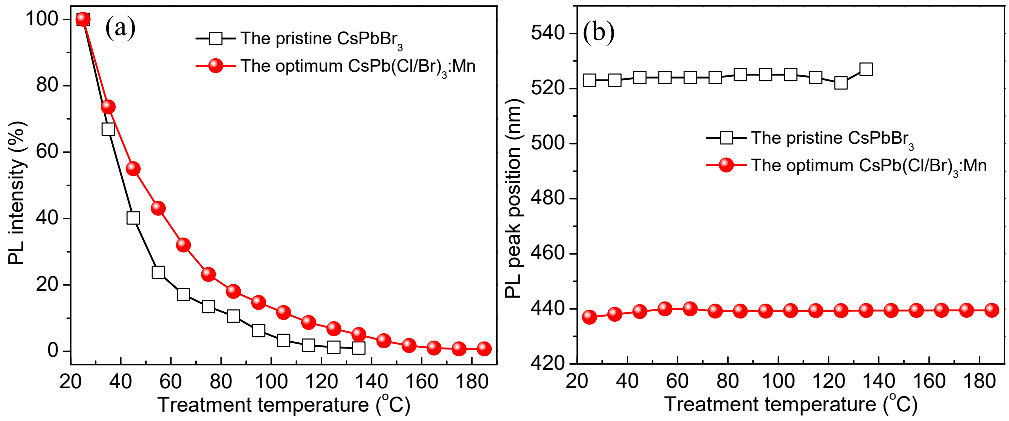

Figure S14. The changes of PL peak (a) intensities and (b) positions of the pristine $CsPbBr_3$ and the optimum $CsPb(Cl/Br)_3$:Mn product as the increase of the treatment temperature.

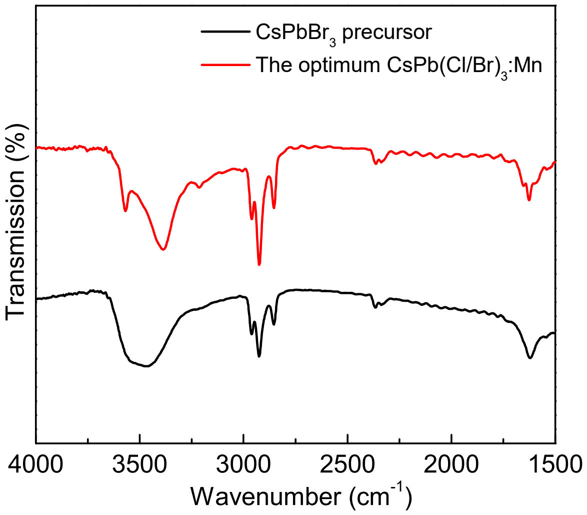

Figure S15. FTIR spectra of the $CsPbBr_3$ precursor and the optimum $CsPb(Cl/Br)_3$:Mn product. The main vibration bands at 3475 and 1625 cm^{-1} are attributed to the symmetric stretching of N-H and asymmetric NH_3^+ deformation [1], respectively. The vibration bands in the range of 2926 and 2856 cm^{-1} are ascribed to the hydrocarbon groups [2].

References

1. Li, X.; Cai, W.; Guan, H.; Zhao, S.; Cao, S.; Chen, C.; Liu, M.; Zang, Z. Highly stable CsPbBr3 quantum dots by silica-coating and ligand modification for white light-emitting diodes and visible light communication. *Chemical Engineering Journal* **2021**, 419, 129551, doi:10.1016/j.cej.2021.129551.
2. Chen, W.; Shi, T.; Du, J.; Zang, Z.; Yao, Z.; Li, M.; Sun, K.; Hu, W.; Leng, Y.; Tang, X. Highly Stable Silica-Wrapped Mn-Doped $CsPbCl_3$ Quantum Dots for Bright White Light-Emitting Devices. *ACS Appl. Mater. Interfaces* **2018**, 10, 43978-43986, doi:10.1021/acsami.8b14046.
